# Case report: Clinical and molecular characterization of two siblings affected by Brody myopathy

**DOI:** 10.3389/fneur.2023.1170071

**Published:** 2023-06-02

**Authors:** Daniele Velardo, Sara Antognozzi, Martina Rimoldi, Serena Pagliarani, Filippo Cogiamanian, Sergio Barbieri, Stefania Corti, Giacomo Pietro Comi, Dario Ronchi

**Affiliations:** ^1^Fondazione IRCCS Ca' Granda Ospedale Maggiore Policlinico, Neuromuscular and Rare Disease Unit, Milan, Italy; ^2^Fondazione IRCCS Ca' Granda Ospedale Maggiore Policlinico, Neurology Unit, Milan, Italy; ^3^Dino Ferrari Center, Department of Pathophysiology and Transplantation, University of Milan, Milan, Italy; ^4^Fondazione IRCCS Ca' Granda Ospedale Maggiore Policlinico, Neurophysiology Unit, Milan, Italy

**Keywords:** Brody myopathy, SERCA1, *ATP2A1*, WES, neuromuscular disorder

## Abstract

Exercise-induced muscle stiffness is the hallmark of Brody disease, an autosomal recessive myopathy due to biallelic pathogenic variants in *ATP2A1*, encoding the sarcoplasmic/endoplasmic reticulum Ca^2+^ ATPase SERCA1. About 40 patients have been reported so far. Our knowledge about the natural history of this disorder, genotype–phenotype correlations and the effect of symptomatic treatment is partial. This results in incomplete recognition and underdiagnosis of the disease. Here, we report the clinical, instrumental, and molecular features of two siblings presenting childhood-onset exercise-induced muscle stiffness without pain. Both the probands display difficulty in climbing stairs and running, frequent falls, delayed muscle relaxation after exertion. Cold temperatures worsen these symptoms. No myotonic discharges were observed at electromyography. Whole Exome Sequencing analysis in the probands revealed the presence of two *ATP2A1* variants: the previously reported frameshift microdeletion c.2464delC and the likely pathogenic novel splice-site variant c.324 + 1G > A, whose detrimental effect was demonstrated in *ATP2A1* transcript analysis. The bi-allelic inheritance was verified by Sanger sequencing in the unaffected parents. This study expands the molecular defects associated with Brody myopathy.

## 1. Introduction

Brody Myopathy (BM, MIM # 601003) is a muscle disorder characterized by childhood onset exercise-induced progressive impairment of muscle relaxation, stiffness, cramps, and myalgia, predominantly in upper and lower limbs and face (eyelids). Symptoms generally improve after a few minutes of rest and may be exacerbated by cold. This disorder is recessively inherited and associated with pathogenic variants in the *ATP2A1* gene encoding for the Sarco(Endoplasmic) Reticulum Calcium ATPase protein SERCA1 (1–3).

SERCA1 catalyzes the ATP-dependent uptake of Ca^2+^ from the cytosol to sarcoplasmic reticulum taking part in the regulation of calcium levels in the sarcoplasmic reticulum and therefore muscle contraction (4, 5). In Brody myopathy patients, the activity of SERCA1 in type II muscle fibres is reduced, resulting in delayed muscle relaxation, silent cramps, muscle weakness and muscle atrophy. The reduction of SERCA1 activity has been documented in available muscle samples from affected patients, supporting at biochemical and histochemical level the diagnosis of Brody myopathy. Since SERCA1 is uniquely expressed in type II (fast-twitch) skeletal muscle fibres, exercise-induced muscle stiffness in Brody disease is primarily triggered by phasic (rapid and intense contractions), but not tonic (slow movements), activity (3).

Although about 40 patients from 28 different families affected with Brody myopathy have been reported so far, since the first description in 1969 (6), our knowledge about disease progression and pathogenesis are far from being exhaustive (2, 4, 7–12).

Here we report two siblings with clinical symptoms suggestive of Brody myopathy. Whole Exome Sequencing analysis (WES) allowed the identification of two *ATP2A1* variants, one of which was novel. Clinical, instrumental, and molecular findings are discussed in view of previous literature in the field.

## 2. Case reports

### 2.1. Patient 1

**Figure 1:**
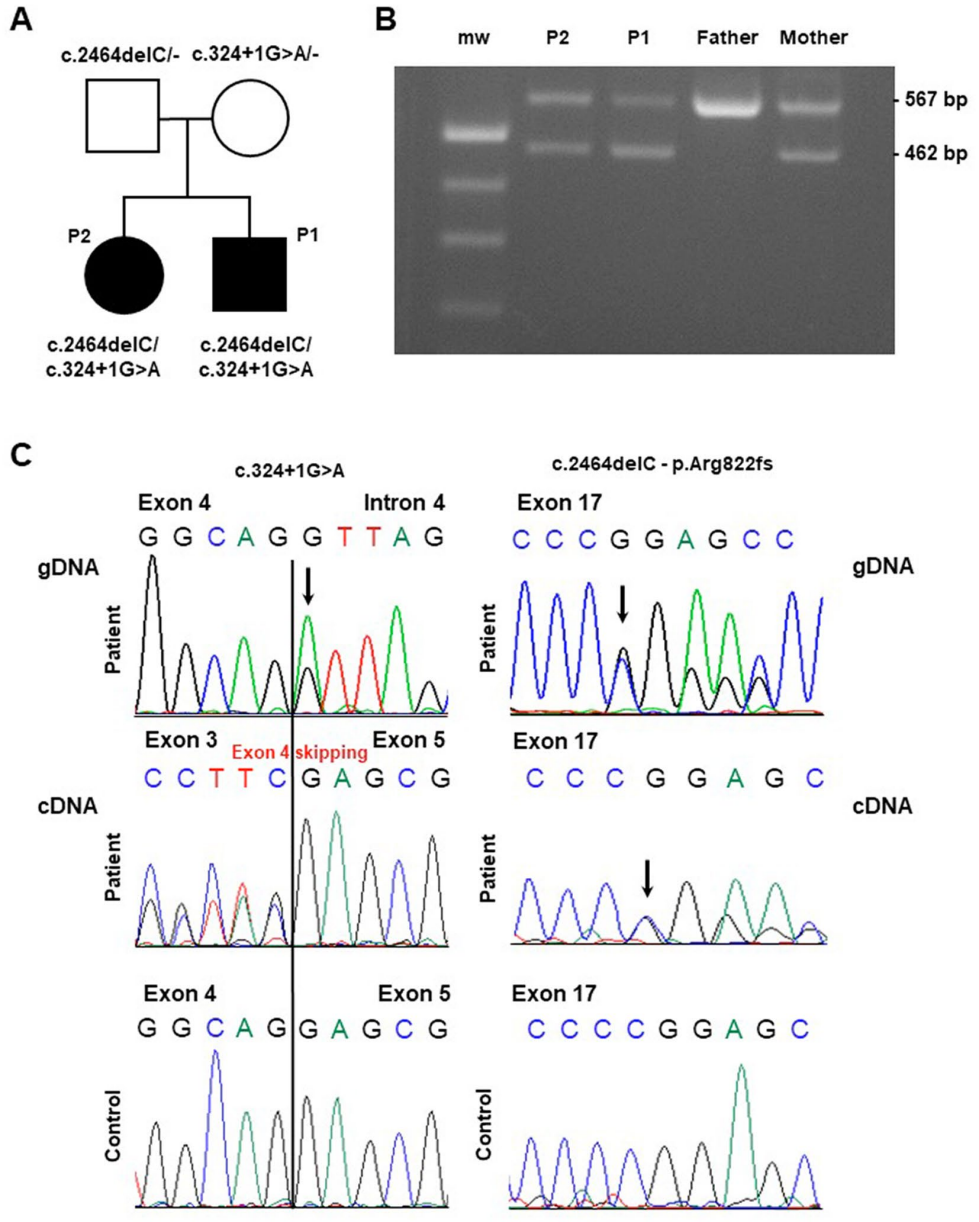
Molecular findings in the investigated pedigree. **(A)** Pedigree of the family. Black symbols indicate the affected probands (patient 1: “P1” and patient 2: “P2”) carrying the described *ATP2A1* variants. **(B)** Gel agarose showing RT-PCR products encompassing exons 1 and 6, obtained by blood extracted cDNA showing, besides the wild-type band, a smaller fragment compatible the skipping of part of the *ATP2A1* transcript in the affected siblings and their mother (“mw” indicates the molecular weight). **(C)** Sequence electropherogram of genomic (gDNA) and complementary (cDNA) DNA products showing the effect of the c.324 + 1G > A variation in the alteration of physiological splicing of *ATP2A1*. The heterozygous deletion c.2464delC, resulting in the loss of *ATP2A1* reading frame, is observed at genomic and cDNA level.

Patient 1 is a 19-years-old boy, second-born to non-consanguineous and healthy parents of Italian origin. Family history is uneventful and negative for neuromuscular problems (see Figure 1).

The prenatal and perinatal history is unremarkable. His psychomotor development was regular.

Since his first childhood, he showed difficulty climbing and descending stairs and running, with frequent falls. At the age of 5 years, he experienced transient upper limb tremors with spontaneous resolution (Table 1).

**Table 1:** Patients timelines.

Patient 1				
2008 Childhood	2015 12 yo	January 2022 18 yo + 1 mo	May 2022 18 yo + 5 mos	October 2022 18 yo + 10 mos
First signs and symptoms	1st neurological evaluation; *SLC2A1* seq. (neg)	Referral to our Institute and clinical evaluation; *SNC4A* seq. (neg)	CK dosage (normal); EMG and S/L exercise tests; Informed consent for WES	WES confirms the diagnosis of BM

Patient 2				
2005 Childhood	2015 15 yo	January 2022 22 yo	May 2022 22 yo + 4 mos	October 2022 22 yo + 9 mos
First signs and symptoms	1st neurological evaluation; NGS (metabolic diseases) (neg); CK dosage (normal); EMG	Referral to our Institute and clinical evaluation	EMG and S/L exercise tests; Informed consent for WES	WES confirms the diagnosis of BM

NGS, next generation sequencing; WES, whole exome sequencing; yo, years old; mo/mos, month/months; neg, negative; seq., sequencing; S/L, short/long.

At the age of 12, a neurological examination revealed mild running impairment and delayed muscle relaxation after closing fists. His serum creatine kinase (CK) levels were normal. In addition, to exclude a GLUT1 deficiency syndrome with infantile onset, he underwent *SLC2A1* gene sequencing, which was negative.

At the age of 18 years, the proband was referred to our center for neurological examination.

The clinical evaluation showed a negative neurological examination, except for an awkward running with lower limbs muscle stiffness. After repeated movements, such as fist opening and closing, wrist and ankle flexion and extension, delayed muscle relaxation phenomenon became evident bilaterally.

Electromyography (EMG) and nerve conduction studies (NCS) results were normal. In addition, a short and long exercise test on bilateral deep finger flexor muscles was performed, which was unremarkable. Specifically, both before and after repeated voluntary contractions, in basal condition and after limb cooling, there were no myotonic discharges, however the patient clinically demonstrated delay of muscle relaxation after repetitive efforts (silent contractures).

During the diagnostic assessment at our Center, the patient underwent *SCN4A* gene sequencing to exclude a Non-Dystrophic Myotonia (NDM, specifically the Paramyotonia Congenita of von Eulenburg, MIM #168300). This analysis turned out negative.

The patient currently reports a feeling of muscle stiffness during exercise, not accompanied by pain, which forces him to stop and rest to resume the physical activity; he does not report cramps, nor myoglobinuria. He regularly practices sports activity.

Notably, proband’s symptoms worsen upon exposure to cold temperatures but are stable over time.

### 2.2. Patient 2

Patient 2 is Patient 1’s 23 years old sister. She was born by induced delivery at 30 weeks of gestation after spontaneous pregnancy complicated by gestosis. Perinatal period was characterized by two episodes of pneumothorax. Her psychomotor development was regular.

Clinical symptoms, including age and onset modalities, overlap those observed in her brother (Table 1).

At the age of 15, she was referred to another Institute for neurological evaluation: the CK dosage and NCS/EMG analysis were normal. After genetic counseling, she underwent a Next Generation Sequencing (NGS) analysis targeted on genes related to metabolic conditions, which gave negative results. A carb-free diet was started with no benefit.

Lastly, she was referred to our Institute at the age of 22 years. Her neurological evaluation was overlapping with her brother’s. The NCS/EMG was again normal; the short and long exercise test performed on deep finger flexor muscles and on quadriceps, before and after effort, revealed silent contractures (prolonged involuntary muscle contractions) following voluntary phasic contractions, without electrical activity detected by needle.

To date, she reports difficulties in walking, running, and climbing stairs. She complains of a sensation of motor impairment as soon as the muscle activity begins (muscle stiffness at the start of exercise). She currently practices physical activity.

## 3. Methods

### 3.1. DNA analysis

After obtaining signed written informed consent, genomic DNA was extracted from peripheral blood obtained from the patients and their parents, on a QiaSymphony Automated Nucleic Acid Extraction Platform (QIAGEN).

Whole Exome Sequencing was performed starting from 100 ng of high-quality DNA of the two affected siblings and the parents by using Agilent SureSelectXT Human All Exon V8 library preparation and target enrichment kit. The libraries underwent paired-end sequencing on a NextSeq2000 Illumina platform. The variants included in the generated VCF files were annotated (according to the genome assembly of hg19) and classified according to an internal analysis pipeline (13) and by using the bioinformatic tool eVai Expert Variant Interpreter v2.7.

Filtering criteria applied were: (i) variants with a Quality Score > 30, (ii) variants with allele frequency < 0.1 (gnomAD), (iii) variants shared by affected subjects, (iv) variants presenting biallelic inheritance, and (v) variants presenting a coding impact or in conserved splice sites. The candidate variants in the *ATP2A1* gene were validated by using PCR amplification followed by Sanger sequencing (Thermo Fisher Big Dye Terminator v3.1) on an ABI Prism 3,130 automated DNA analyzer.

### 3.2. Transcript analysis

Total RNA was extracted from peripheral blood obtained from the subjects investigated by using Thermo Fisher Tempus Spin RNA Isolation Kit. RNA was retrotranscribed into cDNA using Maxima Reverse Transcriptase (Thermo Scientific). For the analysis of *ATP2A1* transcript, specific primers were designed to amplify the regions corresponding to exons 1–6 and 17 (available upon request) based on the reference sequence NM_ 004320. Resulting Polymerase Chain Reaction (PCR) products were stained with ethidium bromide and visualized on 2% agarose gels and then were sequenced as described above.

## 4. Results

Whole Exome Sequencing analysis identified two *ATP2A1* (NM_004320.6) variants in the affected siblings: the microdeletion chr16:28913640C/− corresponding to c.2464delC (p.Arg822Glyfs*49) and the single nucleotide variant chr16:28892341G/A corresponding to c.324 + 1G > A. The two variants segregated from father and mother, respectively (see Figure 1A).

The c.2464delC variant is rare (GnomAD 2.1 frequency in non-Finnish European population is 0.003%) and has been previously reported as a likely pathogenic variant in the ClinVar database (Accession VCV000464084.5).

The c.324 + 1G > A variant is not reported in population databases. This variant, affecting the conserved donor splice site downstream Exon 4, is predicted to alter physiological splicing. By using Reverse Transcription PCR analysis on proband’s blood-extracted RNA, we observed the skipping of the whole Exon 4 as demonstrated by gel agarose electrophoresis (Figure 1B) and sequencing (Figure 1C). This alteration is expected to preserve the reading frame. Both the variants are classified as Likely Pathogenic (class 4), according ACMG criteria (PVS1, PM2) (14).

## 5. Discussion

Here we report the clinical and molecular features of two siblings in which a diagnosis of Brody myopathy was clinically suspected and confirmed by Whole Exome Sequencing applied to the patients pedigree. These findings were compared with those reported in a recent publication investigating a cohort of 40 patients affected with Brody disease (7).

BM is known to typically manifest in the first decade of life, even though patients usually do not present to a physician until their third decade (7).

In our patients, the first evidence of exercise induced muscle stiffness occurred during childhood. Anyway, the siblings were referred to our Institute for neurological examination and received a molecular diagnosis only at the age of 18 (patient 1) and 22 (patient 2). Among the probands to date reported, only 7 patients have been diagnosed before age 22, even though symptoms had emerged relatively early, during childhood or before the age of 10 years.

Both our patients suffer from exercise-induced muscle stiffness with delayed muscle relaxation, predominantly affecting the lower limbs, with symptoms emerging after explosive, short, and repetitive contractions: this is coherent with the selective type II muscle fiber involvement usually observed in BM. The high percentage of type II muscle fibres in orbicularis oculi muscle also explains the eyelid involvement reported in 63% of probands. Anyway, facial muscles, and eyelids were preserved in our patients.

Similarly to the majority of Brody myopathy patients to date reported (25 out of 36, 70%), our patients mention an increase of symptoms upon exposure to cold temperatures.

Both our probands can currently practice sports (including strength exercises) regularly. On the Modified Rankin Scale (MRS), we could assign a patients disability score of 1/5, corresponding to “no significant disability despite symptoms, able to perform all usual activities and tasks.” Our patients do not need any symptomatic treatment and they never underwent physical therapy. In addition, their symptoms do not show a progression and are stable over time. Considering the 40 BM patients so far reported, symptoms were progressive in 23% of them but did not become debilitating: the MRS was performed in 23 of them and scored “1” and “2” (mild disability: no longer able to carry out all the previous activities but independent in walking/daily life activities) in 10 (43%) and 13 probands (57%), respectively (7).

Regarding the EMG findings, none of the patients previously described had myotonic discharges on EMG, while silent contractures were present in 18/28 patients (64%) (7). Even in our two patients, the EMG was normal and short and long exercise tests showed silent contractures.

Except for the EMG findings, the symptoms described in BM are commonly reported in many other neuromuscular disorders, so that Brody myopathy might resemble other hereditary conditions genetically determined, such as myotonic dystrophies or sodium/chloride channelopathies. For this reason, Patient 1 underwent *SCN4A* gene sequencing at the age of 18, since a Paramyotonia Congenita of von Eulenburg was suspected (2, 15–19).

In inherited neuromuscular disorders, a comprehensive clinical assessment contributes to achieve a molecular diagnosis (i.e., by addressing the most appropriate genetic testing). This concept is easily applied to Brody disease where the following cardinal clinical features are associated with the disorder: muscle stiffness consequent to short, repetitive, and explosive movements, the presence of symptoms manifesting on vigorous exercise, the absence of a warm-up phenomenon (2, 19).

WES analysis, our current choice for the diagnosis of familial forms of neuromuscular presentations, revealed the presence of the previously unreported c.324 + 1G > A variant in *ATP2A1.* This variant is classified as likely pathogenic and results in the skipping of Exon 4 coding for a region encompassing SERCA1 transmembrane domain M2. This region helps to anchor the actuator domain to the rest of the protein (1), and its absence likely results in protein instability and impairment of SERCA1 function. The second variant, c.2464delC, has been previously reported in independent ClinVar submissions associated with Brody Myopathy. No obvious correlation between genotype and phenotype has so far emerged.

To date, there is no specific treatment and cure for BM. Some therapeutic strategies have been considered such as the use of drugs promoting Ca^2+^ efflux from the cytosol or aiming to reduce proteasomal SERCA1 disposal (20, 21).

In conclusion, our report expands the clinical and molecular features associated with *ATP2A1* variants in Brody Myopathy. Our findings contribute to define the clinical presentation associated with this condition.

## Data availability statement

The datasets presented in this article are not readily available because of ethical and privacy restrictions. Requests to access the datasets should be directed to the corresponding author.

## Ethics statement

The studies involving human participants were reviewed and approved by Comitato Etico Milano Area 2 Fondazione IRCCS Ca' Granda Ospedale Maggiore Policlinico (Milan, Italy). The patients/participants provided their written informed consent to participate in this study. Written informed consent was obtained from the individual(s) for the publication of any potentially identifiable images or data included in this article.

## Author contributions

DV, MR, and DR interpreted the results, conceived the idea, revised the literature, and wrote the manuscript. SA and SP performed genetic analysis. DV, MR, and SC made the clinical evaluation. FC and SB performed neurophysiological assessment. SC and GPC performed a critical revision of the manuscript for important intellectual content. All authors contributed to the article and approved the submitted version.

## Funding

This study was (partially) funded by Italian Ministry of Health – Current research IRCCS Ca′ Granda Ospedale Maggiore Policlinico and by SEQMD project (IRCCS Cà Granda Ospedale Maggiore Policlinico, PI: Giacomo Comi).

## Conflict of interest

The authors declare that the research was conducted in the absence of any commercial or financial relationships that could be construed as a potential conflict of interest.

## Publisher’s note

All claims expressed in this article are solely those of the authors and do not necessarily represent those of their affiliated organizations, or those of the publisher, the editors and the reviewers. Any product that may be evaluated in this article, or claim that may be made by its manufacturer, is not guaranteed or endorsed by the publisher.
